# Ginkgolide A Ameliorates LPS-Induced Inflammatory Responses In Vitro and In Vivo

**DOI:** 10.3390/ijms18040794

**Published:** 2017-04-10

**Authors:** Yan Li, Yannan Wu, Xinlei Yao, Fang Hao, Chunlei Yu, Yongli Bao, Yin Wu, Zhenbo Song, Ying Sun, Lihua Zheng, Guannan Wang, Yanxin Huang, Luguo Sun, Yuxin Li

**Affiliations:** 1 Institute of Genetics and Cytology, Northeast Normal University, Changchun 130024, China; liy848@nenu.edu.cn (Y.L.); yaoxl613@nenu.edu.cn (X.Y.); yucl885@nenu.edu.cn (C.Y.); wuy705@nenu.edu.cn (Y.W.); zhenglh015@nenu.edu.cn (L.Z.); wanggn258@nenu.edu.cn (G.W.); 2 National Engineering Laboratory for Druggable Gene and Protein Screening, Northeast Normal University, Changchun 130024, China; wuyn712@nenu.edu.cn (Y.W.); haof991@nenu.edu.cn (F.H.); baoyl800@nenu.edu.cn (Y.B.); songzb484@nenu.edu.cn (Z.S.); suny040@nenu.edu.cn (Y.S.); huangyx356@nenu.edu.cn (Y.H.)

**Keywords:** Ginkgolide A, inflammation, AMPK, NF-κB, MAPKs

## Abstract

Ginkgolide A (GA) is a natural compound isolated from *Ginkgo biloba* and has been used to treat cardiovascular diseases and diabetic vascular complications. However, only a few studies have been conducted on the anti-inflammatory effects of GA. In particular, no related reports have been published in a common inflammation model of lipopolysaccharide (LPS)-stimulated macrophages, and the anti-inflammatory mechanisms of GA have not been fully elucidated. In the present study, we extensively investigated the anti-inflammatory potential of GA in vitro and in vivo. We showed that GA could suppress the expression of pro-inflammatory mediators (cyclooxygenase-2 (COX-2) and nitric oxide (NO) and pro-inflammatory cytokines (tumor necrosis factor (TNF)-α, interleukin (IL)-6 and IL-1β) in LPS-treated mouse peritoneal macrophages, mouse macrophage RAW264.7 cells, and differentiated human monocytes (dTHP-1) in vitro. These effects were partially carried out via downregulating Nuclear factor kappa-B (NF-κB), Mitogen-activated protein kinases (MAPKs) (p38 mitogen-activated protein kinase and extracellular signal-regulated kinase (ERK), but not c-Jun N-terminal kinase (JNK), and activating the AMP-activated protein kinase (AMPK) signaling pathway also seems to be important. Consistently, GA was also shown to inhibit the LPS-stimulated release of TNF-α and IL-6 in mice. Taken together, these findings suggest that GA can serve as an effective inflammatory inhibitor in vitro and in vivo.

## 1. Introduction

Inflammation is a complex pathological process which results from tissue injury or infection [1]. It is an important defensive immune response with health benefits [2]. However, acute and chronic inflammatory states can lead to serious conditions, such as asthma, arthritis, inflammatory bowel disease, atherosclerosis, and cancer [3]. Multiple types of immune cells are involved in inflammatory responses. Macrophages, as primary immune cells, play a key role in homeostasis and defense, particularly in the initial stage of the inflammatory process [4] and the modulation of host defense mechanisms via the production of pro-inflammatory mediators, such as nitric oxide (NO) and cytokines [1].

Lipopolysaccharide (LPS), the primary component of the outer membrane of Gram-negative bacteria, stimulates the activity of immune cells and triggers an inflammatory response against bacterial invasion [4,5]. Therefore, stimulation of macrophages by LPS has been widely used to establish models for exploring inflammation-related mechanisms and anti-inflammatory drug discovery and development. LPS stimulation can cause excessive release of pro-inflammatory mediators in macrophages through activation of nuclear factor-kappa B (NF-κB) and mitogen-activated protein kinase (MAPK) signaling pathways [6,7]. Prostaglandins and NO are generated enzymatically by cyclooxygenase-2 (COX-2) and nitric oxide synthases (NOS), respectively, and are critical pro-inflammatory mediators produced by LPS-stimulated macrophages [8]. Activated macrophages can also secrete many inflammatory cytokines, including tumor necrosis factor (TNF)-α, interleukin (IL)-6, and IL-1β [9]. The elevation of inflammatory cytokine levels is closely linked to the development of inflammation-associated diseases such as atherosclerosis [2].

Currently available anti-inflammatory drugs, such as steroids and nonsteroidal agents, always have various adverse effects with long-term use. Therefore, developing anti-inflammatory drugs with greater efficacy and minimal toxicity remains a challenge [10]. Towards this aim, we established a firefly luciferase reporter screening system driven by the *IL-6* gene promoter to identify new anti-inflammatory candidates (unpublished data). Via screening over 300 natural compounds, ginkgolide A (GA) was identified to clearly inhibit the activity of the *IL-6* gene promoter and was further confirmed to suppress IL-6 expression in our preliminary research (unpublished data). GA is a lactone extracted from *Ginkgo biloba*, one of the leading herbal medicines used to treat various diseases that has been shown to have notable antioxidant and free radical scavenging activity [11]. Although other types of ginkgolides have shown anti-inflammatory activity, which of GA has not been widely reported. Only two studies recently showed that GA could mediate protection against liver inflammation in CCl_4_-induced mice [12] and reduce inflammatory responses in high-glucose-stimulated human umbilical vein endothelial cells [13]. However, the exact anti-inflammatory activity of GA and the underlying mechanisms have not been fully elucidated. In particular, its anti-inflammatory effects in LPS-stimulated macrophages have not been reported thus far.

Based on our previous preliminary reports, this study was conducted to extensively explore the anti-inflammation capacity of GA and clarify the underlying molecular mechanism by using LPS-stimulated macrophages in vitro, as well as in vivo, in the mouse model. Our findings suggest that GA can be an effective candidate for use in the treatment of inflammation.

## 2. Results

### 2.1. GA Inhibits LPS-Stimulated COX-2 Expression and NO Production

**Figure 1:**
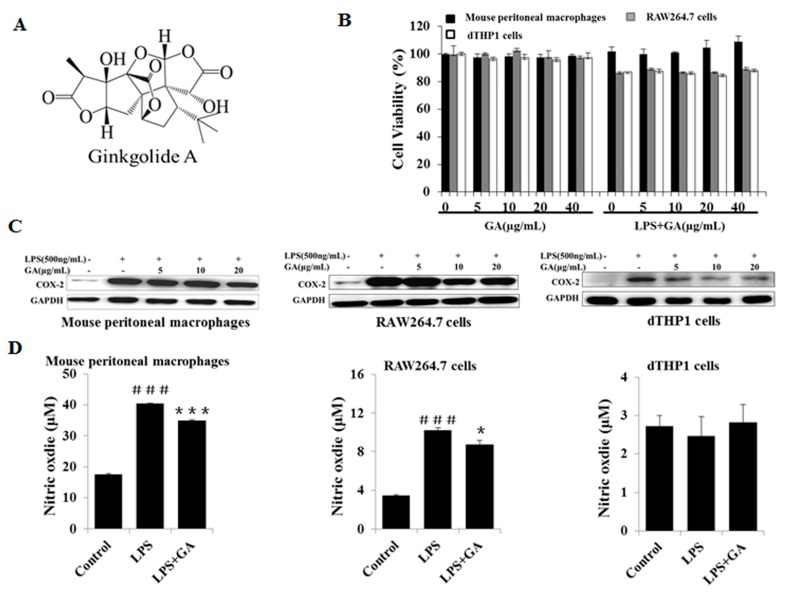
GA inhibits LPS-stimulated COX-2 expression and NO production. (**A**) Chemical structure of GA; (**B**) The effects of GA on cell viability. Macrophages were treated with different concentrations of GA (5, 10, 20, and 40 μg/mL) in the absence or presence of 500 ng/mL LPS for 24 h. Cell viability was quantified with the MTT assay; (**C**) The effects of GA on the expression of COX-2. Following pre-treatment with GA for 1 h, macrophages were co-incubated with or without 500 ng/mL LPS for 24 h. The protein expression of COX-2 was determined by Western blotting; and (**D**) the effects of GA on NO production. Macrophages were treated as in (**B**). Production of NO in the culture medium was measured by the Griess reaction assay. GA, Ginkgolide A; LPS, lipopolysaccharide; dTHP-1, differentiated THP-1; COX-2, cyclooxygenase-2; NO, nitric oxide; MTT, 3-(4,5-dimethylthiazol-2-yl)-2,5-diphenyltetrazolium bromide; GAPDH, glyceraldehyde-3-phosphate dehydrogenase. Experiments were repeated three times. ^###^ *p* < 0.001 versus the control group; * *p* < 0.05; *** *p* < 0.05 versus the LPS-treated group.

The 3-(4,5-dimethylthiazol-2-yl)-2,5-diphenyltetrazolium bromide (MTT) assay was used to determine whether GA (Figure 1A) could influence the cell viability of macrophages. GA alone, or GA co-treatment with 500 ng/mL LPS, showed no cytotoxic effect at concentrations from 0 to 40 μg/mL for 24 h in mouse peritoneal macrophages, RAW264.7 and differentiated THP-1 (dTHP-1) cells (Figure 1B). Based on these results, GA was used in all subsequent experiments at a concentration range of 0–20 μg/mL.

COX-2 is an important inflammatory mediator, which serves as a rate-limiting enzyme to catalyze the conversion of arachidonic acid to prostaglandins. COX-2 has been shown to be rapidly induced in LPS-activated macrophages [14], consistent with our study. GA was found to suppress COX-2 expression in LPS-stimulated mouse peritoneal macrophages, RAW264.7, and dTHP1 cells in a dose-dependent manner (Figure 1C). NO is another important inflammatory mediator, which is involved in regulating many physiological processes, including neurotransmission, vascular relaxation, platelet aggregation, and the immune response [4]. The exposure of macrophages to LPS in this study resulted in a significant secretion of NO after 24 h of incubation, while GA was found to downregulate NO production in LPS-stimulated mouse peritoneal macrophages and RAW264.7 cells. However, we did not observe the same effect in dTHP-1 cells, since 500 ng/mL of LPS failed to induce NO production in our experiment (Figure 1D). The above data suggested that GA could inhibit LPS-induced expression of the inflammatory mediators COX-2 and NO in macrophages to some degree.

### 2.2. GA Inhibits LPS-Stimulated Expression and Production of Pro-Inflammatory Cytokines in Macrophages

**Figure 2:**
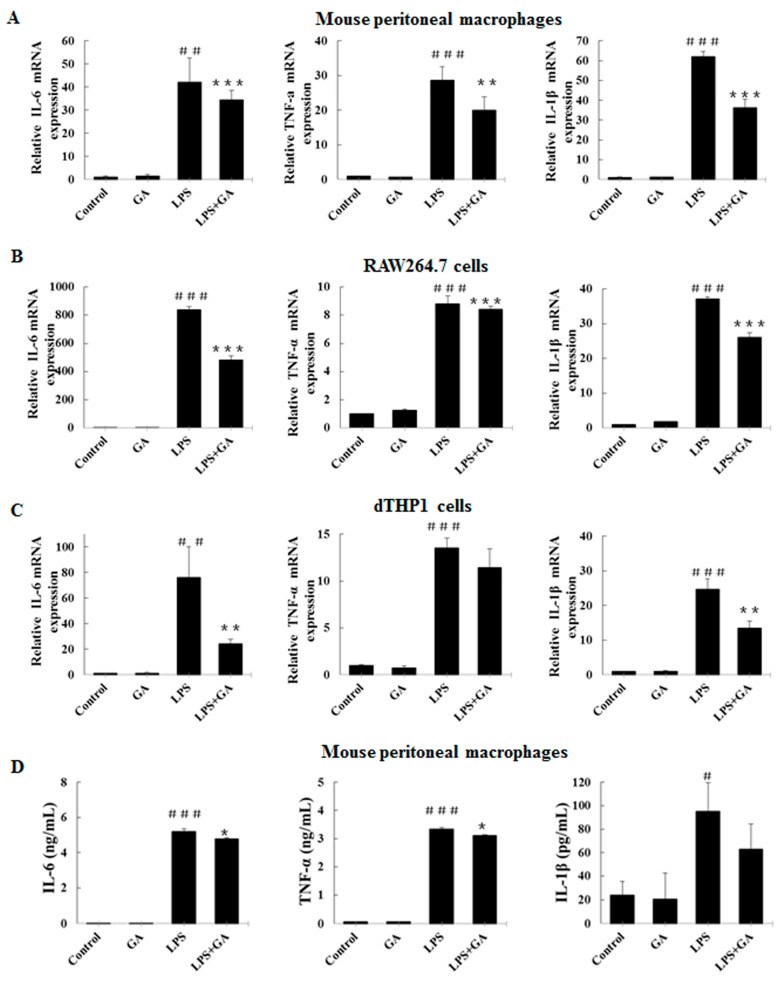

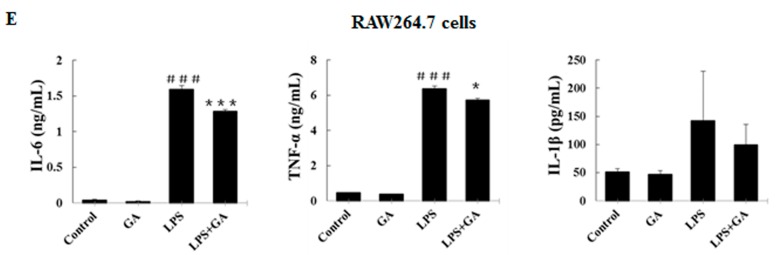
GA inhibits LPS-stimulate expression and production of pro-inflammatory cytokines in macrophages. (**A**–**C**) Effects of GA on LPS-stimulated mRNA expression of *TNF-α*, *IL-6*, and *IL-1β*. Following pre-treatment with GA for 1 h, mouse peritoneal macrophages (**A**), RAW264.7 cells (**B**), and dTHP-1 cells (**C**) were co-incubated with or without 500 ng/mL LPS for 10 h. The total RNA was prepared, and the mRNA expression levels of *IL-6*, *TNF-α*, and *IL-1β* were determined by RT-qPCR. (**D**,**E**) Effects of GA on LPS-stimulated production of TNF-α, IL-6, and IL-1β. Following pre-treatment with GA for 1 h, mouse peritoneal macrophages (**C**) and RAW264.7 cells (**D**) were co-incubated with or without 500 ng/mL LPS for 24 h. Levels of IL-6, TNF-α, and IL-1β in culture media were quantified by ELISA. TNF, tumor necrosis factor; IL, interleukin; RT-qPCR, quantitative Real-Time RT-PCR; ELISA, enzyme-linked immunosorbent assay. Experiments were repeated three times. ^#^ *p* < 0.05, ^##^ *p* < 0.01, ^###^ *p* < 0.001 versus the control group; * *p* < 0.05; ** *p* < 0.01; *** *p* < 0.05 versus the LPS-treated group.

To further evaluate the anti-inflammatory activity of GA on LPS-stimulated macrophages, we examined its effect on LPS-stimulated expression of pro-inflammatory cytokines, such as IL-6, IL-1β, and TNF-α. First, we tested mRNA levels of cytokines using quantitative Real-Time RT-PCR (RT-qPCR). As shown in Figure 2A–C, LPS could dramatically stimulate the mRNA transcription of IL-6, IL-1β, and TNF-α in all three types of macrophages tested, while pre-treatment with GA greatly diminished the stimulatory effects of LPS. We then examined the production of cytokines in the supernatant of macrophages by enzyme-linked immunosorbent assay (ELISA). Consistently, upon pre-treatment with GA, the LPS-stimulated production of pro-inflammatory cytokines IL-6 and TNF-α was found to be reduced dramatically in mouse peritoneal macrophages and RAW264.7 cells. Although IL-1β levels in the LPS + GA group consistently showed a tendency of reduction compared with the LPS control group, they failed to reach statistical significance (Figure 2D,E). Furthermore, GA treatment alone minimally affected the basal level of mRNA expression and the production of these cytokines (Figure 2). These results indicated that GA has significant anti-inflammatory potential by suppressing LPS-stimulated pro-inflammatory cytokine expression.

### 2.3. GA Suppresses LPS-Induced Activation of NF-κB and MAPK Signaling

**Figure 3:**
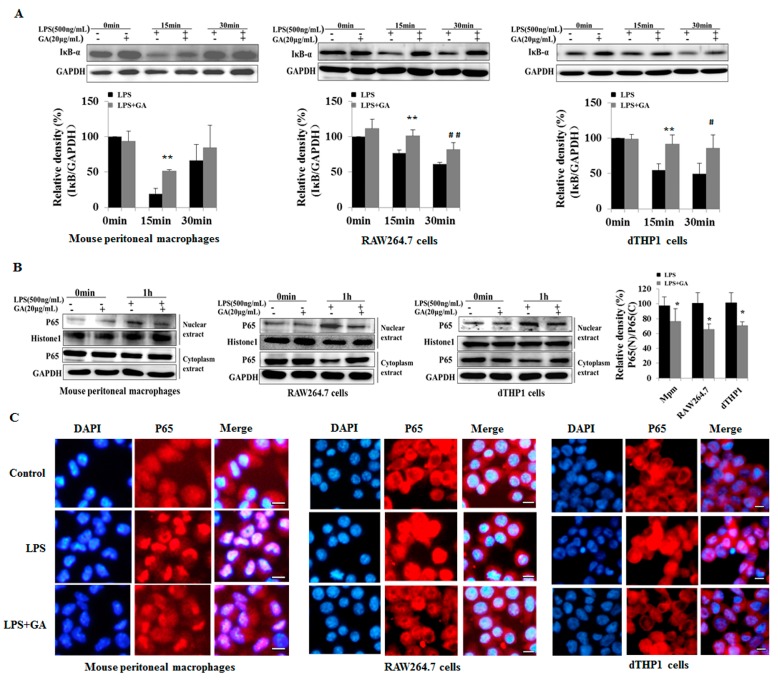
GA suppresses LPS-induced activation of NF-κB signaling. (**A**) Effects of GA on degradation of IκB. Mouse peritoneal macrophages (**left**), RAW264.7 cells (**middle**), and dTHP-1 cells (**right**) were pre-treated with 20 μg/mL GA for 1 h and then stimulated with LPS (500 ng/mL) for 0, 15, and 30 min. Total cellular lysates were prepared and analyzed for IκB by Western blotting. The histograms below the blots show the relative density ratios (fold changes) measured by Image J. Values are presented as means ± SD of three independent experiments. ** *p* < 0.01 versus the LPS group at 15 min; ^#^ *p* < 0.05, ^##^ *p* < 0.01 versus the LPS group at 30 min. (**B**,**C**) Effects of GA on nuclear translocation of p65; (**B**) mouse peritoneal macrophages (**left**), RAW264.7 cells (**middle**), and dTHP-1 cells (**right**) were pre-treated with 20 μg/mL GA for 1 h and then stimulated with LPS (500 ng/mL) for 1 h. Nuclear and cytosolic extracts were isolated, and the levels of p65 in each fraction were determined by Western blotting. Histone 1 was detected as a nuclear internal control, and GAPDH was used as a cytosolic internal control. The histogram shows relative density ratios (fold changes) measured by Image J. Values are presented as means ± SD of three independent experiments * *p* < 0.01 versus the LPS group. Mpm, mouse peritoneal macrophages; (**C**) Mouse peritoneal macrophages (**left**), RAW264.7 cells (**middle**), and dTHP-1 cells (**right**) were treated as in (**B**). The nuclear translocation of p65 was determined by immunofluorescence staining. DAPI-stained nuclei are indicated by blue fluorescence. Nuclear and cytosolic p65 are indicated by red fluorescence. Nuclei appear purple in merged images. Bar, 50 μm. NF-κB, nuclear factor kappa-B; IκB, inhibitor of NF-κB; DAPI, 2-(4-Amidinophenyl)-6-indolecarbamidine dihydrochloride.

LPS is well known to trigger serial signal transduction events including the activation of NF-κB and MAPKs (Extracellular signal regulated kinase (ERK), c-Jun N-terminal kinase (JNK), and p38 mitogen-activated protein kinase) [15,16]. Subsequently, the activation of these proteins can regulate the production of pro-inflammatory cytokines. Upon LPS stimulation, inhibitor of NF-κB (IκB) is degraded to release NF-κB p65 into the nucleus to drive the expression of target genes. In our study, under the condition of LPS stimulation, IκB started to degrade rapidly at 15 min, and then p65 translocated into the nucleus. When macrophages were pre-treated with GA, the degradation of IκB was suppressed at 15 min and 30 min (Figure 3A), and the subsequent translocation of cytosolic NF-κB p65 into the nucleus was suppressed as well (Figure 3B). Quantification of the immunoblots showed that the average inhibitory effects of GA on degradation of IκB were 25.19%, 22.72%, and 36.53% and on the translocation of p65 into the nucleus were 20.81%, 34.67%, and 30.79%, respectively, in LPS-stimulated mouse peritoneal macrophages, RAW264.7 cells and dTHP-1 cells. Distributions of p65 (red fluorescence) in the nucleus and cytoplasm were evaluated by the immunofluorescence assay in all three types of macrophages. In agreement with the above results, upon LPS stimulation, GA suppressed the translocation of cytosolic NF-κB p65 into the nucleus (Figure 3C). These results suggested that GA suppressed NF-κB signaling in LPS-treated macrophages.

**Figure 4:**
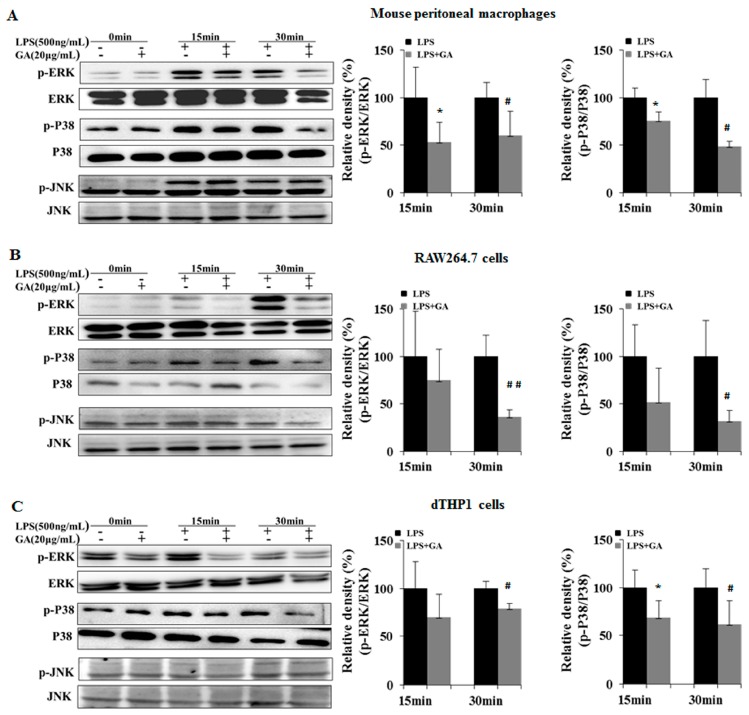
GA suppresses activation of MAPKs. (**A**) Mouse peritoneal macrophages; (**B**) RAW264.7 cells; and (**C**) dTHP-1 cells were pre-treated with 20 μg/mL GA for 1 h and then stimulated with LPS (500 ng/mL) for 0, 15, and 30 min. Total cellular proteins were obtained from the cells and analyzed for phosphorylation of ERK, JNK, and p38 by Western blotting. The histograms show relative density ratios (fold changes) measured by Image J. Values are presented as means ± SD of three independent experiments. * *p* < 0.05 versus LPS group at 15 min; ^#^ *p* < 0.05, ^##^ *p* < 0.01 versus LPS group at 30 min. p, phospho; ERK, extracellular signal-regulated kinase; JNK, c-Jun N-terminal kinase.

MAPKs are considered important pathways which mediate secretion of LPS-stimulated inflammatory factors [17]. Likewise, upon LPS stimulation, the phosphorylation of MAPKs (ERK, JNK, and p38) were remarkably heightened at 15 or 30 min. GA suppressed the LPS-stimulated phosphorylations of ERK and p38 to different extents, whereas JNK phosphorylation was scarcely influenced (Figure 4A–C). Quantification of immunoblots showed that the average inhibitory effects of GA on phospho-ERK were 43.13%, 46.04%, and 23.8% ,and on phospho-p38 levels were 35.07%, 53.42%, and 34.68%, respectively, in LPS-stimulated mouse peritoneal macrophages, RAW264.7 cells, and dTHP-1 cells.

### 2.4. GA Upregulates AMPK Signaling

**Figure 5:**
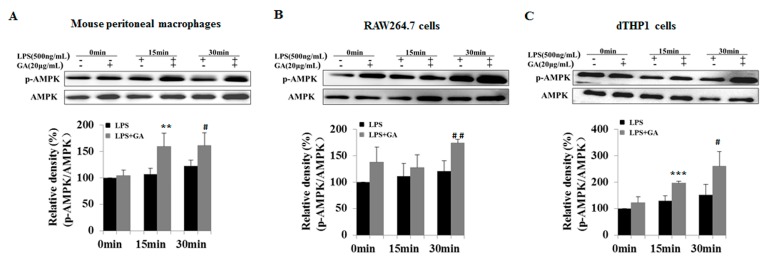
GA upregulates AMPK signaling in LPS-stimulated macrophages. (**A**) Mouse peritoneal macrophages; (**B**) RAW264.7 cells; and (**C**) dTHP-1 cells were treated with the same method as in Figure 4. Cell lysates were prepared and analyzed for phosphorylation of AMPK by Western blotting. The histograms below the blots show relative density ratios (fold changes) measured by Image J. Values are presented as means ± SD of three independent experiments. ** *p* < 0.01; *** *p* < 0.001 versus LPS group at 15 min; ^#^ *p* < 0.05, ^##^ *p* < 0.01 versus LPS group at 30 min. AMPK, AMP-activated protein kinase.

AMP-activated protein kinase (AMPK) is known as a key regulator of cellular energy homeostasis. Indeed, AMPK activators have been associated with inflammation [18,19]. Here, we sought to further determine whether GA influences the AMPK pathway, Upon LPS stimulation, GA was found to upregulate phosphorylation of AMPK in the three types of macrophages (Figure 5). These results suggested that GA could ameliorate the LPS-stimulated inflammatory response by suppressing the NF-κB and MAPK (ERK and p38) pathways, while promoting the AMPK pathway. From the relative changes of phospho-AMPK, the average activation effects of GA were 39.9%, 29.9%, and 62.25%, respectively, in LPS-stimulated mouse peritoneal macrophages, RAW264.7 cells, and dTHP-1 cells.

### 2.5. GA Inhibits NF-κB and MAPks Pathway through AMPK Activation

**Figure 6:**
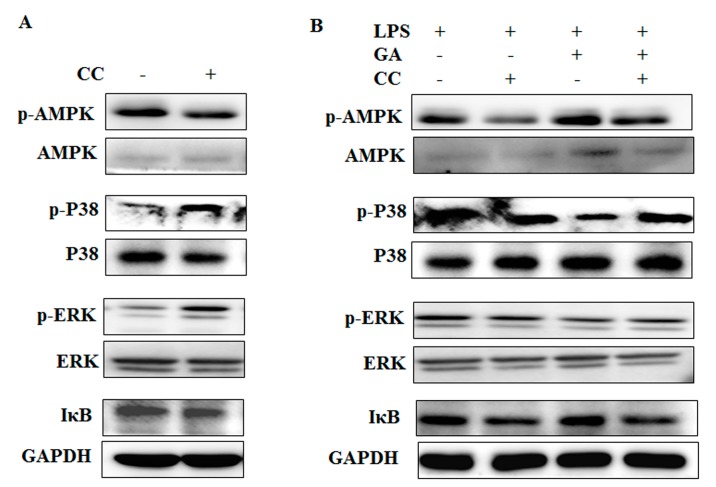
GA inhibits NF-κB and MAPK pathways through AMPK activation in LPS-stimulated mouse peritoneal macrophages. Mouse peritoneal macrophages were pre-treated with 12.5 μM CC for 1 h, followed by GA (20 μg/mL) treatment for 1 h, and LPS (500 ng/mL) stimulation for 0 min (**A**) or 30 min (**B**) subsequently. Total cellular proteins were obtained and subjected to Western blotting to detect the phosphorylation of AMPK, ERK, and p38 and the degradation of IκB. CC, compound C.

We speculated that activation of the AMPK pathway might regulate the activation of MAPKs and NF-κB pathways based on previous reports [20,21]. To test this possibility, we used the AMPK inhibitor Compound C (CC) to pretreat mouse peritoneal macrophages. CC was observed to effectively downregulate the phosphorylation of AMPK and simultaneously enhance phosphorylation of ERK, p38, and the degradation of IκB (Figure 6A). These results suggest that indeed crosstalk does occur between AMPK and the MAPK and NF-κB pathways. We further tested the effects of CC on GA inhibition of LPS activation of MAPK and NF-κB pathways. As shown in Figure 6B, in the presence of GA plus LPS, CC could partially reverse the inhibitory effect of GA on NF-κB and MAPK activation. These results indicate that GA might elicit its inhibitory effects on LPS-stimulated activation of pro-inflammatory pathways at least in part through activation of the AMPK pathway.

### 2.6. GA Attenuates the Inflammatory Response in LPS-Treated BALB/c Mice

**Figure 7:**
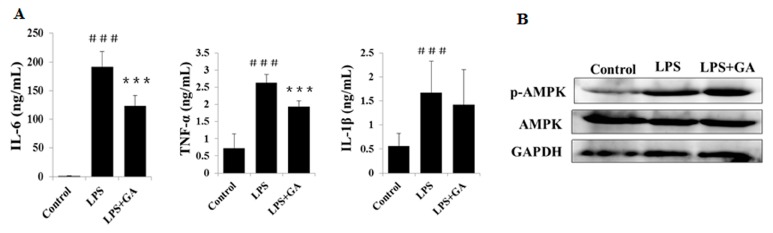
GA attenuates inflammatory response in LPS-treated BALB/c mice. (**A**) Effects of GA on production of TNF-α, IL-6, and IL-1β in LPS-induced BALB/c mice. BALB/c mice were randomly divided into three groups. The control group was administered the same amount of solvent i.p. (*n* = 5). The LPS-induced group was administered LPS (250 μg/kg) (*n* = 6). The treatment group was administered LPS (250 μg/kg) and GA (20 mg/kg) at the same time (*n* = 5). Two hours after the injection, blood was harvested, and serum was separated to measure IL-6 (**left**), TNF-α (**middle**) and IL-1β (**right**) levels by ELISA. ^###^ *p* < 0.001 versus control group; *** *p* < 0.05 versus LPS-treated group. (**B**) The effects of GA on the AMPK pathways in LPS-treated BALB/c mice. BALB/c mice were treated with the same method as in (**A**). Blood samples were treated with red blood cell lysis buffer to harvest immune cells for analysis of AMPK phosphorylation by Western blotting.

LPS has been established as an agent for mimicking the initial clinical features of systemic inflammation [7]. Thus, we further investigated the therapeutic efficacy of GA in a systemic model of inflammation induced using 250 μg/kg LPS in BALB/c mice. The LPS-stimulated production levels of IL-6, IL-1β, and TNF-α in serum were upregulated dramatically when compared to the control group. When treated with 20 mg/kg GA, levels of IL-6 and TNF-α were significantly suppressed, while that of IL-1β was little affected in this study (Figure 7A). In addition, we harvested immune cells from the blood to investigate the potential impact of GA on the AMPK pathway in vivo. Indeed, GA could heighten further the phosphorylation of AMPK in the context of LPS stimulation (Figure 7B). Together, these results showed that GA could exert anti-inflammatory effects both in vitro and in vivo.

## 3. Discussion

IL-6 plays important roles during inflammation and is involved in the pathogenesis of different inflammatory diseases [22]. TNF-α has a central role in initiating and regulating the release of adhesion molecules, and IL-1β mediates inflammatory responses at the local and systemic levels during inflammatory responses [23]. NO and COX-2 are involved in the pathogenesis of inflammatory diseases [8]. These inflammatory mediators are produced by activated macrophages and amplify the inflammatory response [24]. In the present study, we took advantage of the *IL-6* gene promoter to establish a firefly luciferase reporter screening system and identified GA as a strong suppressor of *IL-6* promoter activity. GA was further demonstrated to not only be able to inhibit IL-6 expression, but it was also shown to have universal inhibitory effects on the production of other pro-inflammatory mediators to different extents in macrophages of three different origins. Moreover, the same inhibitory effects were also observed in LPS-treated mice. Our results indicate that GA could potently restrain macrophage-mediated inflammation stimulated by LPS. Taken together with recent reports showing that GA reduced inflammatory responses in CCl_4_-induced liver [13] and high-glucose-stimulated human umbilical vein endothelial cells [12], all of these findings corroborate the global anti-inflammatory potential of GA targeting versatile phlogogenous factors.

In addition, our study provides novel insight into the possible mechanisms underlying the anti-inflammatory effect of GA. We demonstrated that GA could clearly restrict LPS-stimulated activation of the NF-κB pathway via blocking LPS-triggered IκB degradation and translocation of p65 in tested macrophages. The NF-κB pathway is one of the most important pathways that mediate inflammatory responses including transcriptional control of the expression of many pro-inflammatory mediators. Our observation is in agreement with a prior study in which GA was shown to inhibit activation of the NF-κB pathway to mediate the protection of mice against CCl_4_-induced liver inflammation [12]. In addition to the NF-κB pathway, LPS also induces inflammation through activating MAPK pathways. Upon activation of MAPKs, transcription factors are activated to trigger the expression of target genes including those of pro-inflammatory mediators [6]. In this study, we further showed that GA pre-treatment significantly suppressed LPS-stimulated phosphorylation of p38 and ERK, but not JNK in macrophages, which may also contribute to the anti-inflammatory effect of GA. The regulatory effects of GA on MAPK pathways in macrophages have not been reported before. Moreover, AMPK has been reported to act as a potent negative regulator of the NF-κB pathway and pro-inflammation, and its activation is directly associated with reduced IκB degradation [16]. Here, for the first time, GA was shown clearly to enhance the activation of AMPK in LPS-stimulated macrophages, as well as in immune cells of LPS-treated BALB/c mice. In addition, the AMPK inhibitor Compound C could partially abolish the inhibitory effect of GA on NF-κB and MAPK activation. These findings suggest that GA exerts anti-inflammatory effects by enhancing activation of AMPK to counter LPS-stimulated activation of the NF-κB and MAPK pathways. As AMPK is proposed to be a key regulator of cellular and many therapeutic agents, such as metformin used in the treatment of diabetes and atherosclerosis through activation of AMPK [25,26], GA may potentially be used to treat these diseases through this mechanism as well.

*G. biloba* is a unique woody species widely available in China, which has broad applications due to its medicinal value such as the treatment of cerebrovascular dysfunctions and peripheral vascular disorders. Ginkgolides, as one of the main active ingredients in *G. biloba*, are known as potent inhibitors of platelet activating factor. Among the different types of ginkgolides (A, B, C, J, and M) [27], GB has been extensively studied, including its anti-inflammatory effects [15,28]. However, GA, as the chemical analog of GB, has not received much attention, and the studies on GA have mainly focused on the treatment of cardiovascular diseases and diabetic vascular complications. Only a few recent studies have explored the anti-inflammatory effects of GA, but its role specifically in macrophages is rarely reported. Here, GA was demonstrated to have strong anti-inflammatory activity, which may mechanistically supply the molecular basis for its application in the treatment of cardiovascular diseases and diabetic vascular complications, since these two types of conditions are closely associated with chronic inflammation. These findings also imply that GA would have a wide range of potential therapeutic applications, including those that target inflammatory disorders. Our findings also provide additional evidence to support *G. biloba* as a valuable medicinal herb.

## 4. Materials and Methods

### 4.1. Mice

Pathogen-free female BALB/c mice and C57BL/6J mice (18–22 g, 7–10 weeks) were purchased from the Experimental Animal Center, Medical College of Norman Bethune, Jilin University. On arrival, mice were acclimatized prior to experimentation for at least one week. The mice were maintained in a 12 h light/dark cycle with free access to food and water. Animal experiments were approved by the Ethics Committee of Northeast Normal University (Approval No: NENUKJ-16-020, Approval Date: 9 May 2016) and were performed in accordance with the Regulations for the Administration of Affairs Concerning Experimental Animals approved by the State Council and promulgated by Decree No. 2 of the State Science and Technology Commission (1988).

### 4.2. Reagents and Antibodies

GA (HPLC ≥ 97%) was purchased from the National Institute for the Control of Pharmaceutical and Biological Products (Beijing, China). Antibodies specific for p38, JNK, histone 1and COX-2 were provided by Santa Cruz Biotechnology (Santa Cruz, CA, USA). Antibodies specific for IκB-α, p65, p-p38, p-ERK, ERK, p-JNK, p-AMPK, and AMPK were obtained from Cell Signaling (Beverly, MA, USA). Antibody against GAPDH (KC-5G4) was provided by KangChen Bio-tech (Shanghai, China). Phorbolmyristate acetate (PMA), MTT and LPS were purchased from Sigma-Aldrich (St. Louis, MO, USA). Immunofluorescence Staining Kit with Cy3-labeled goat anti-rabbit IgG, DAPI, CC (6-1-3-pyridin-4-ylpyrazolo-1-pyrimidine), NO kit, and enhanced chemiluminescence (ECL) reagent were obtained from Beyotime (Shanghai, China). Mouse ELISA kits for IL-6, TNF-α and IL-1β were obtained from R & D Systems (Beijing, China).

### 4.3. Cell Culture

RAW264.7 cells (mouse macrophages) were cultured in Dulbecco’s Modified Eagle’s medium (DMEM) with 100 U/mL penicillin and 100 µg/mL streptomycin sulfate. THP-1 cells (human leukemia monocytes) were grown in Roswell Park Memorial Institute (RPMI) 1640 medium with 0.05 mM 2-mercaptoethanol and supplemented with 0.2 μg/mL PMA to induce THP-1 into dTHP-1 for 24 h. Both types of cells were supplemented with heat-inactivated 10% fetal bovine serum (FBS) and cultured in a 5% CO_2_ incubator at 37 °C. Mouse peritoneal macrophages were acquired from ascites of C57BL/6J mice, which were injected intraperitoneally (i.p.) with 1 mL of 6% soluble starch for three consecutive days before sacrifice. Cells were cultured for 2 h and then washed twice with PBS to remove adherent cells. The mouse peritoneal macrophages were then incubated in DMEM with heat-inactivated 10% FBS in a 5% CO_2_ incubator at 37 °C.

### 4.4. Cell Viability

The cytotoxicity of GA on mouse peritoneal macrophages, RAW264.7 cells, and dTHP1 cells was assessed by the MTT assay. Briefly, macrophages were seeded in 96-well plates (5 × 10^4^ cells/well) overnight and then incubated with different concentrations of GA (5, 10, 20, and 40 μg/mL) after pre-treatment for 1 h with or without 500 ng/mL LPS for 24 h. Thereafter, 20 μL MTT (2.5 mg/mL) was added to each well and co-cultured for 4–6 h. Subsequently, the medium was discarded, and 100 μL DMSO was added to dissolve the formazan crystals. Optical densities were measured at 490 nm using a spectrophotometric plate reader (Bio-Rad, Hercules, CA, USA). The experiment was repeated three times.

### 4.5. RT-qPCR

Macrophages were seeded in six-well plates and treated with GA in the absence or presence of 500 ng/mL LPS for 10 h. The total RNA of macrophages was extracted with Trizol reagent (Invitrogen, Carlsbad, CA, USA) according to the manufacturer’s instructions and reverse transcribed into cDNA using a real-time PCR kit (TransGen Biotech, Beijing, China). RT-qPCR was performed using the SYBR Green I PCR master mix kit (TAKARA, Beijing, China). The amplification protocol was carried out as follows: 95 °C for 10 min, followed by 40 cycles of 95 °C for 10 s, 60 °C for 1 min, and 60 °C for 30 s. The PCR primers for specific genes are shown in Table 1.

### 4.6. ELISA

Culture media or sera were harvested to measure levels of IL-6, TNF-α and IL-1β using commercial ELISA kits (R & D Systems, Beijing, China) according to the manufacturer’s instructions.

### 4.7. NO Assay

Culture medium (50 μL) was collected and mixed with Griess reagent (Beyotime, Shanghai, China), and the optical density was read at 540 nm on a spectrophotometric plate reader. The NO concentration in the culture supernatants was estimated against a standard curve, which was calibrated using sodium nitrite standards.

### 4.8. Western Blot Analysis

Total proteins and cytosolic-nuclear proteins were extracted as previously described [29]. The extracts were electro-blotted onto a polyvinylidene fluoride (PVDF) membrane following separation by 12% sodium dodecyl sulfate polyacrylamide gel electrophoresis (SDS-PAGE). The PVDF membrane was incubated with blocking solution with 5% skim milk for 2 h at room temperature, followed by incubation with a specific antibody overnight at 4 °C and then with horseradish peroxidase-conjugated secondary antibodies for 1 h at room temperature. Finally, blots were visualized by ECL.

### 4.9. Fluorescence Microscopy for Visualization of NF-κB p65 Localization

Macrophages were seeded in 96-well plates overnight. After pre-treatment with GA for 1 h with or without 500 ng/mL LPS for 1 h, the cells were washed and fixed with 4% paraformaldehyde for 20 min, permeabilized with 0.2% Triton X-100 for 10 min, and then blocked with 5% BSA for 30 min at room temperature. Subsequently, cells were incubated with an anti-P65 antibody overnight at 4 °C, washed and then incubated with secondary antibodies conjugated with fluorophores Cy3 at room temperature for 1 h. After being washed with PBS, cells were then treated with DAPI for nuclear staining. DAPI and P65 subunit stainings were observed with a fluorescent light microscope.

### 4.10. LPS-Induced Mice

BALB/c mice were randomly divided into three groups. Mice of the control group were administered the same amount of solvent by i.p. injection. Systemic inflammation was induced in the LPS group by i.p. administration of LPS (250 μg/kg). The treatment group was administered LPS (250 μg/kg) and GA (20 mg/kg) at the same time. Two hours after the injection, blood was harvested from the orbital plexus, and the serum was separated to measure levels of IL-6, IL-1β, and TNF-α using ELISA kits.

### 4.11. Statistical Analysis

All experiments were performed in triplicate, and the statistical significance of the experimental results was calculated by the Student’s *t*-test. The statistical significance was set as * *p* < 0.05, ** *p* < 0.01, *** *p* < 0.001, **^#^** *p* < 0.05, **^##^** *p*< 0.01, and **^###^** *p* < 0.001. Error bars denote the ± standard deviation (SD).

## 5. Conclusions

In the present study, we found that GA could inhibit LPS-mediated inflammation in three types of macrophages, due at least in part to its regulation of NF-κB, MAPKs (ERK and p38), and AMPK pathways in vitro. As a consequence, pro-inflammatory mediators, including NO, COX-2, and pro-inflammatory cytokines, such as TNF-α, IL-1β, and IL-6, were downregulated. Consistently, GA could suppress TNF-α and IL-6 production in LPS-stimulated BALB/c mice, as well. Therefore, GA may be developed as a potential treatment option for inflammatory diseases.

## Figures and Tables

**Table 1:** Mouse (M) and human (H) primers for RT-qPCR.

Gene	Forward Primer	Reverse Primer
*IL-6(M)*	5′-TCCAGTTGCCTTCTTGGGAC-3′	5′-GTGTAATTAAGCCTCCGACTTG-3′
*IL-6(H)*	5′-GTGAAAGCAGCAAAGAGGCACT-3′	5′-ACCAGGCAAGTCTCCTCATTGA-3′
*TNF-α(M)*	5′-GACCCTCACACTCAGATCATCTTCT-3′	5′-CCTCCACTTGGTGGTTTGCT-3′
*TNF-α(H)*	5′-TCAATCGGCCCGACTATCTC-3′	5′-CAGGGCAATGATCCCAAAGT-3′
*IL–1β(M)*	5′-TCGTGCTGTCGGACCCATAT-3′	5′-GTCGTTGCTTGGTTCTCCTTGT-3′
*IL–1β(H)*	5′-GCACGATGCACCTGTACGAT-3′	5′-AGACATCACCAAGCTTTTTTGCT-3′
*GAPDH(M)*	5′-AAATGGTGAAGGTCGGTGTG-3′	5′-TGAAGGGGTCGTTGATGG-3′
β-actin(H)	5′-CGTGCGTGACATTAAGGAGAAG-3′	5′-GGAAGGAAGGCTGGAAGAGTG-3′

## Abbreviations

AMPK	AMP-activated protein kinase
COX-2	Cyclo-oxygenase-2
CC	Compound C
ERK	Extracellular regulated kinases
GA	Ginkgolide A
JNK	c-Jun N-terminal kinases
LPS	Lipopolysaccharide
MAPKs	Mitogen-activated protein kinases
NO	Nitric oxide
NF-κB	Nuclear factor kappa-B
